# Characterisation of Cooked Cheese Flavour: Non-Volatile Components

**DOI:** 10.3390/foods12203749

**Published:** 2023-10-12

**Authors:** Rosa C. Sullivan, Samantha Nottage, Fiyinfolu Makinwa, Maria Jose Oruna-Concha, Colette C. Fagan, Jane K. Parker

**Affiliations:** 1 Department of Food and Nutritional Sciences, University of Reading, Whiteknights, Reading RG6 6DZ, UKfmakinwa@outlook.com (F.M.); m.j.oruna-concha@reading.ac.uk (M.J.O.-C.);; 2 Synergy Flavours Ltd., Hillbottom Road, Sands Industrial Estate, High Wycombe HP12 4HJ, UK

**Keywords:** cooked cheese, tastant, diketopiperazine, γ-glutamyl-dipeptide, cheddar, mozzarella, parmesan, organic acid, sugar, reduced fat

## Abstract

This work examined the role of selected non-volatile compounds in cooked cheese flavour, both as tastants and as precursors of aroma generation in the Maillard reaction. The effect of cooking on the concentration of selected non-volatile compounds (organic acids, sugars, amino acids, γ-glutamyl dipeptides, and diketopiperazines) in six cheeses (mature Cheddar, mozzarella, Parmesan, and mild Cheddar (low, medium, and high fat)) was determined. Sugars, amino acids, and γ-glutamyl dipeptides were extracted and analysed by LC, whereas diketopiperazines were extracted by solid-phase extraction and analysed by GC-MS. Sugars, amino acids, and γ-glutamyl dipeptides decreased in concentration during cooking, whereas diketopiperazines and some organic acids increased in concentration. Diketopiperazines were above the taste threshold in some cooked cheeses and below the threshold in uncooked cheeses. The role of fat content in cooked cheese flavour is discussed. Furthermore, γ-glutamyl dipeptide concentration increased during 24 months of ageing in low, medium, and high-fat Cheddars, with similar levels of γ-glutamyl dipeptide detected in aged low and high-fat Cheddars. This work will give valuable insight for the dairy industry to inform the development of cheeses, especially low-fat variants, for use in cooked foods.

## 1. Introduction

Cheese is a major commodity produced by the dairy industry globally. Mintel [1] estimated the UK market value for cheese to be £3.2 billion in 2020. Applications for cheese include a variety of cooked dishes, such as toppings for pasta and pizza, grilled or melted (e.g., fondue). Previous research into cheese flavour has focused on uncooked cheese, and little is known about the effect of cooking on the taste of cheese.

The aim of this study was to determine the effect of cooking on the concentration of selected non-volatile compounds in a range of popular cheeses in the UK (Parmesan, mature Cheddar, mozzarella, low-fat mild Cheddar, medium-fat mild Cheddar, and high-fat mild Cheddar). Analytes were selected based on their contribution to taste in uncooked cheese and their potential role as precursors in the Maillard reaction, an important pathway for the formation of flavour and colour when foods are cooked. Five groups of analytes were chosen for analysis: amino acids, sugars, organic acids, γ-glutamyl peptides, and diketopiperazines (DKPs). A future study focuses on volatile compounds in cooked cheese.

It is hypothesised that amino acids, peptides, and sugars in cheese decrease in concentration during cooking due to participation in the Maillard reaction. Furthermore, some organic acids (e.g., acetic acid) and DKPs are hypothesised to increase in concentration due to their formation during cooking. We hypothesise that these changes may be substantial enough to alter the balance of suprathreshold tastants in cooked cheese compared to uncooked cheese.

Organic acids, especially lactic acid, are key to the characteristic sharpness and low pH of uncooked cheese [2]. Amino acids possess various taste properties, including bitterness, sweetness, sourness, and umami. In particular, glutamic acid has been shown to contribute significantly to umami flavour in cheeses [2,3,4]. The most abundant sugars in cheese are lactose and its component monosaccharides, glucose and galactose. While these sugars contribute to sweetness in most dairy products, the majority of the lactose in milk is lost to the whey during cheesemaking; therefore, lactose concentrations are below the sweet threshold in most cheeses [2].

γ-Glutamyl dipeptides have been reported in various uncooked cheeses [5,6], where they contribute kokumi taste (mouthfulness). The concentration of γ-glutamyl dipeptides increases during the ageing of gouda [5,6]; however, their formation in low-fat cheeses during ageing has not been studied. For this reason, alongside the study of γ-glutamyl dipeptides in cooked and uncooked high, medium, and low-fat mild Cheddar, each Cheddar was also ripened to 24 months with regular analysis of γ-glutamyl dipeptide concentration during ageing.

DKPs are cyclic dipeptides that contribute to bitter and metallic flavours [7]. DKPs have been reported in several cooked foods, including beef [8], chicken essence [9], cocoa [10], coffee [11], bread [12], and sake [13]. Additionally, they have been reported in uncooked Comté cheese [14], but at subthreshold concentrations. 

Further to the characterization of cooked cheese flavour, this study includes a comparison of low- and high-fat mild Cheddar during cooking. Fat and calorie reduction is an important focus for the food industry, and certain cheese-containing products, such as pizza and cheese-topped ready meals, could benefit from the use of lower-fat cheeses. Fat can act as a precursor during the Maillard reaction and also affect the structural and melt properties of cheeses. For this reason, the fat content of cheese has the potential to influence the development of flavour during cooking. The hypothesis of this study is that fat content influences flavour development during the cooking of cheese.

## 2. Materials and Methods

### 2.1. Materials

γ-Glutamyl dipeptides (ƴ-Glu-Glu, ƴ-Glu-Val, ƴ-Glu-Met, ƴ-Glu-Tyr, ƴ-Glu-Leu and ƴ-Glu-Phe) and DKP standards (c-Leu-Pro, c-Val-Pro, c-Pro-Pro, and c-Ala-Pro) were purchased from Bachem (Bubendorf, Switzerland). All other chemicals were purchased from Merck, Gillingham, UK.

### 2.2. Cheeses

Cheeses used in this study were either manufactured in 2017 in the pilot plant at the University of Reading (see Section 2.3) or purchased from a supermarket in 2018 (mature commercial Cheddar (Ched), fresh mozzarella (Mozz), and Parmesan (Parm). Cheddar, mozzarella, and Parmesan represent a range of cheeses that are used in cooked dishes and vary considerably in terms of maturity. Three Cheddar cheeses of differing fat content—low fat (LF, 2% fat), medium fat (MF, 22% fat), and high fat (HF, 35% fat)—were included to determine the effect of fat content on the formation of non-volatile compounds in cooked cheese and on the formation of amino acids and γ-glutamyl peptides during maturation.

### 2.3. Cheesemaking

Milk was obtained from the University of Reading dairy herd of Holstein Friesians (CEDAR, Arborfield, University of Reading, UK). Their diet (expressed per cow per day) included concentrate blend (9.5 kg), hay (1.0 kg), grass silage (19.0 kg), maize silage (24.0 kg), Trafford Gold (cow feed based on wheat by-products) (4.0 kg), fat (0.1 kg), salt (0.1 kg), limestone flour (0.1 kg), and minerals (0.03 kg). The milk was pasteurised (73 °C for 15 s) using a continuous pasteurizer with plate heat exchangers operating at approximately 300 L/h. The fat was separated from the milk using a disc bowl separator and then recombined to produce standardised milk with fat contents of 0.10%, 2.71%, and 3.9%, respectively, in order to produce Cheddar cheese with low, medium, and high fat contents, respectively.

Cheddar cheese making was carried out in 100 L jacketed cheese vats. Standardised milk (100 L) was heated to 30 °C in cheesemaking vats when 0.1 g/L starter culture R604 (Chr. Hansen, Hungerford, UK) was added. The initial pH of the milk was 6.75 ± 0.01. Once the pH decreased to 6.65 ± 0.02, Chymosin, (0.26 g/L, CHYMAX, Chr. Hansen, UK) was added to initiate coagulation. After 60 min (pH 6.60 ± 0.03), the coagulum was cut. Scalding was then initiated by increasing the temperature to 38 °C over 40 min and holding it at this temperature for 50 min. Following the scalding, the whey was drained from the curd, which was then Cheddared. Once the pH reached 5.30 ± 0.01 (approximately 60 min), the curd was milled and salted (2% *w*/*w*) and then pressed in moulds for 24 h. They were then matured at 8 °C for 3 months (producing mild Cheddars), which were used for analysis. Additionally, small portions of the cheeses were further aged to 6, 9, 12, 18, and 24 months for amino acid and γ-glutamyl peptide analysis. The cheese was cut into pieces (approximately 250 g), vacuum-packed, and stored at −20 °C until use. The final weights of high-, medium-, and low-fat Cheddar were 10.2, 8.8, and 6.2 kg, respectively.

### 2.4. Cooked Cheese Sample Preparation

Grated cheese (50 g) was spread evenly on a glass petri dish (90 mm diameter × 10 mm depth) and baked in a GC Oven (Hewlett-Packard 5890 Series II) at 180 °C for 20 min. The GC oven was used as it offers more precise temperature control than typical domestic ovens. It was then cooled to room temperature, immersed in liquid nitrogen, and ground in a coffee grinder (Quest, Liverpool, UK) to obtain a fine powder.

### 2.5. Diketopiperazine Analysis

#### 2.5.1. Solid Phase Extraction

Cheese (~50 g) was cut into 1 cm^3^ pieces, immersed in liquid nitrogen (BOC, Woking, UK), and ground in a coffee grinder (Quest, Liverpool, UK) to obtain a fine powder. In triplicate, a portion of cheese (uncooked or cooked) (5.0 ± 0.1 g) was spiked with 50 µL of internal standard solution (5-methyl-2-hexanone 0.500% in isopropyl alcohol) and vortexed vigorously with 25 mL of HPLC-grade water for 60 min. The slurry was then centrifuged (Thermo Scientific, Paisley, UK) for 3 min at 2038 g and 15 °C. The supernatant underwent solid-phase extraction using SPE cartridges (Strata-X 33 µm polymeric reversed-phase giga tube, Phenomenex (Torrance, CA, USA)). The SPE cartridge was conditioned under vacuum (approximately 30 kPa) using 5 mL of ethanol followed by 5 mL of HPLC-grade water. The sample was loaded slowly onto the cartridge, rinsed with a further 5 mL of water, and then dried by passing air through the cartridge for 30 s. The sample was then eluted slowly from the cartridge with 5 mL of methyl acetate.

#### 2.5.2. GC-MS Analysis of Diketopiperazines

Analyses were performed on an Agilent 7890-5977A GC-MS system (Agilent, Stockport, UK) equipped with an autosampler (Agilent, Stockport, UK). Each liquid extract (1 µL) was injected in splitless mode onto a DB-FFAP polar column (30 m, 0.25 mm I.D., 0.25 µm film thickness) (Phenomenex, Macclesfield, UK). The oven temperature was 45 °C initially, rising by 4 °C/min to 220 °C, and held for 45 min. Helium was used as the carrier gas at 1.2 mL/min. The mass spectrometer was operated in electron ionisation mode with a source temperature of 230 °C, an ionising voltage of 70 eV, and a scan range from m/z 40 to m/z 300 at 5.3 scans/s. The data were acquired and analysed using Masshunter software (Version 4.5, Agilent, UK). Compounds were identified by comparing their mass spectra and linear retention indices with those of authentic standards.

### 2.6. Sugars Analysis

Cheese (~50 g) was cut into 1 cm^3^ pieces, immersed in liquid nitrogen (BOC, UK), and ground in a coffee grinder (Quest, Liverpool, UK) to obtain a fine powder. In triplicate, a portion of cooked or uncooked cheese (5 ± 0.1 g) was vortexed with a sulfuric acid solution (25 mL, 0.09 N) for 1 h. The slurry was centrifuged (Thermo Scientific, UK) for 10 min at 2038 g. The supernatant was filtered by gravity (Whatman 1 filter paper) and then through a disc syringe filter (Agilent, 0.45µm pore size, 15 mm diameter), then frozen at −20 °C until analysis.

Analysis was performed on an Agilent (UK) 1260 Infinity II LC with an Infinitylab XT MSD. Samples (10 µL) were separated through a Ca-phase ion-exchange column (Agilent, UK, 300 × 7.7 mm Hi-Plex Ca) heated at 80 °C at a flow rate of 0.4 mL/min. The mobile phase was 100% water (LC-MS grade, Sigma Aldrich, Poole, UK). Single-ion-monitoring mode was used for the identification of lactose, glucose, and galactose using the sodiated molecular ion (m/z 365, 203, and 203, respectively), along with a comparison of retention time with authentic standards (Sigma Aldrich). The source fragmentor voltage was 135 V, the capillary voltage was 2000 V, and the nozzle voltage was 1500 V. The gas temperature at the source was 300 °C, and the nebuliser pressure was 30 psi. Quantitation was performed by comparison of MS areas against a calibration curve of standard solutions of concentrations of 0.01–100 mg/L (lactose) or 0.01–10 mg/L (glucose and galactose) in sulfuric acid (0.09 N).

### 2.7. Organic Acids Analysis

The extracts used for sugar analysis were also used for organic acid analysis. Analysis was performed on an Agilent (UK) 1260 Infinity II LC with an Infinitylab XT MSD and a diode array UV detector (Agilent, UK). Samples (20 µL) were separated through an H-phase ion-exchange column (Agilent, UK, 300 × 7.7 mm Hi-Plex H) heated at 65 °C at a flow rate of 0.5 mL/min. The mobile phase was water with 0.01 M sulfuric acid. Each acid was identified by comparing the retention time and MS with those of authentic standards. The MS operated in both positive and negative scan modes between 50 and 250 m/z, with a fragmentor voltage of 135 V, a capillary voltage of 3500 V, and a nozzle voltage of 2000 V. The gas temperature in the source was 300 °C, and the nebuliser pressure was 1.38 bar. The diode array detector operated at wavelengths of 220 and 275 nm. Quantitation was performed by comparing diode array areas against a calibration curve (5 points, 50–1000 mg/L in the extracts) for each sugar.

### 2.8. Amino Acid Analysis

The method described by Toelstede et al. [5] was used to prepare a water-soluble extract of each cheese. The organic acids analysed were citric acid, malic acid, lactic acid, acetic acid, and propanoic acid. The uncooked or cooked cheese (12.5 g) was homogenised using a Phillips (Guildford, UK) hand blender for 2 min with deionised water (50 mL). They were centrifuged in a Sigma 3k10 centrifuge (Sciquip, Newtown, UK) for 20 min at 4 °C and 6654 g. The fat layer was removed, and the supernatant was reserved. Deionised water (50 mL) was added to the remaining protein pellet, and the samples were shaken for 15 min using a Heidolph multi-reax shaker (Heidolph Instruments GmbH and Co., Schwabach, Germany). The samples were centrifuged again under the same conditions as above. Both supernatants were combined, and the pH was adjusted to 4.6 with 98–100% formic acid. It was then centrifuged and filtered under a vacuum. The extracts were freeze-dried and ground into a powder. The freeze-dried samples (200 mg) were rehydrated with 5 mL of water and filtered using a 25 mm, 0.2 μm syringe filter. Samples were prepared for amino acid analysis using the EZ-FAAST system (Phenomenex, UK). Analysis was conducted on an Agilent Technologies 6890N GC system coupled to an Agilent 5975 inert XL Mass Selective Detector. The oven was fitted with a ZBAAA GC column. The injection port was held at 250 °C, and the oven programme was as follows: 30 °C/min ramp from 110–320 °C. The carrier gas was helium at a constant flow rate of 1.1 mL/min.

### 2.9. γ-Glutamyl Peptide Analysis

Water-soluble extracts were prepared as described in Section 2.8. γ-Glutamyl dipeptides (ƴ-Glu-Glu, ƴ-Glu-Val, ƴ-Glu-Met, ƴ-Glu-Tyr, ƴ-Glu-Leu and ƴ-Glu-Phe) were analysed according to a modified method to that described by Toelstede and Hofmann [6]. Aliquots (5 µL) of samples were injected into a triple quadruple mass spectrometer coupled with an Agilent 1260 Infinity HPLC system fitted with a 2.1 × 100 mm, 1.8 µm ZORBAX SB-C18 column (all Agilent, Santa Clara, CA, USA). The mobile phase was comprised of acetonitrile and water, each containing 1% formic acid. The flow rate was 0.2 mL/min, and the solvent ratio of acetonitrile to water was 0:100 initially, increasing to 10:90 by 10 min and finally increasing to 100:0 by 25 min which was the final runtime. The mass spectrometer was operating in positive EI mode, using the following settings: ion spray voltage of 4000 eV, fragmentor voltage of 50 eV, collision energy of 10, source temperature (TEM) of 325 °C, and nitrogen curtain gas (CUR) of 2.42 bar. Multiple-reaction monitoring mode (MRM) was performed using the mass transitions previously reported by Toelstede and Hofmann [5]. Peak areas obtained for corresponding mass traces were compared to those of standard solutions of reference peptides to enable quantitative analysis.

### 2.10. Statistical Interpretation

The concentrations of the non-volatile compounds were analysed by one-way analysis of variance (ANOVA) using XLSTAT statistical and data analysis solution (Addinsoft (2020), New York, NY, USA). For those compounds exhibiting a significant difference in the ANOVA, the Fisher’s least significant difference (LSD) test was applied to determine which sample means differed significantly (*p* = 0.05).

## 3. Results

### 3.1. Diketopiperazines

**Figure 1:**
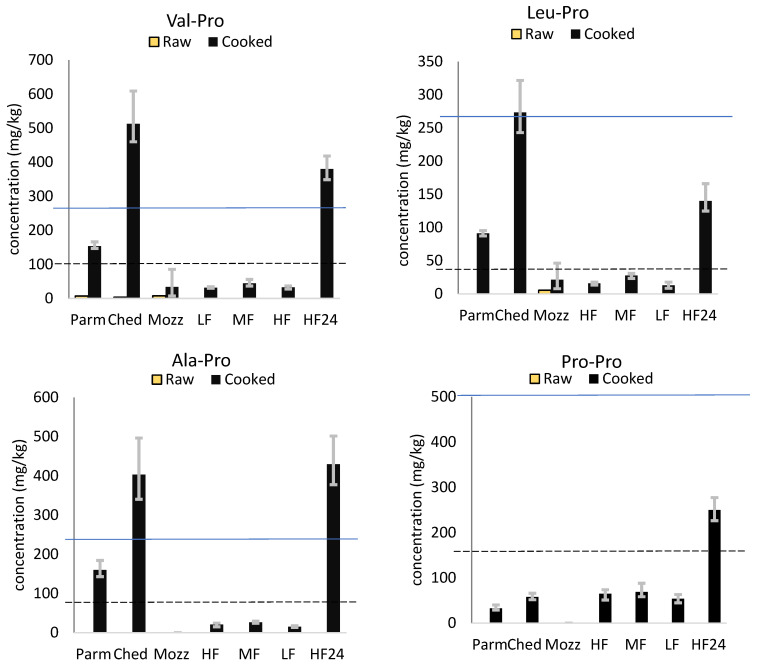
Concentration (mg/kg) of four proline-containing DKPs (mean N = 3) in Parmesan (Parm), mature Cheddar (Ched), mozzarella (Mozz), low-fat 3-month-aged Cheddar (LF), medium fat 3-month-aged Cheddar (MF), high fat 3-month-aged Cheddar (HF), and 24-month-aged high-fat Cheddar (HF24), cooked (black bars) and uncooked (yellow bars). Data are given on a wet weight basis. Black dashed line-metallic taste threshold [10]. Blue solid line-bitter taste threshold [10]. Error bars show minimum and maximum range values.

Figure 1 (Table S1) shows the concentration of four DKPs detected in the cooked and uncooked cheeses on a wet weight basis, along with their metallic and bitter taste thresholds [10]. All DKPs detected were subthreshold in the uncooked cheeses. This agrees with a previous study, which showed that DKPs are present at subthreshold concentrations in uncooked comté and do not contribute to bitter taste [14].

However, the concentrations of DKPs increased significantly (5–150 fold higher) during cooking. Furthermore, some DKPs were detected in the cooked cheeses, which were not detected in their uncooked counterparts. DKPs were present above their bitter taste thresholds in the cooked Ched and HF24 samples and above their metallic thresholds in Parm.

Regarding the role of fat concentration, the DKPs were all below bitter threshold in the three mild Cheddars, although c-Leu-Pro was above the metallic threshold in MF. There were no significant differences in DKP concentration between the HF, MF, and LF Cheddars, and no trends were observed between fat content and DKP formation.

The observation that the Ched (commercially purchased mature Cheddar) contained significantly more DKPs when cooked than any of the mild Cheddars in this study suggested that the ageing period of Cheddar may be correlated with DKP formation when cooked. Uncooked and cooked samples of the HF Cheddar matured for 24 months were also tested for DKP concentration. DKPs were significantly higher in the cooked HF24 than in HF and were similar in concentration to those detected in cooked Ched.

As DKPs were not detected in uncooked HF24, this suggests that an increased ageing period led to the formation of precursors for DKP formation in uncooked Cheddar. DKP formation occurs from small peptides during thermal processing [7] and, more specifically, proline-containing DKPs form from di- and tripeptides with proline in the second position from the N-terminal [15]. The formation of DKPs from di- and tripeptides has been shown to occur both in the presence and absence of glucose [16]. The process of proteolysis during cheese ripening generates small-chain peptides and amino acids from cheese proteins [17]. It is likely that the formation of peptides, which are precursors of DKPs, occurs during Cheddar ripening.

### 3.2. Free Amino Acids

**Figure 2:**
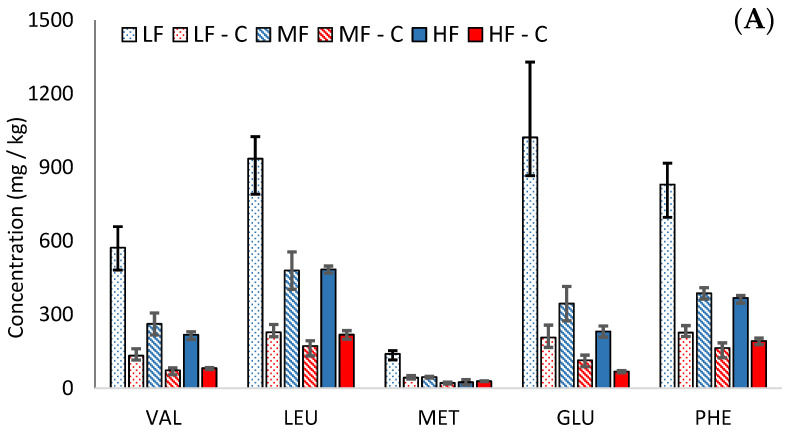

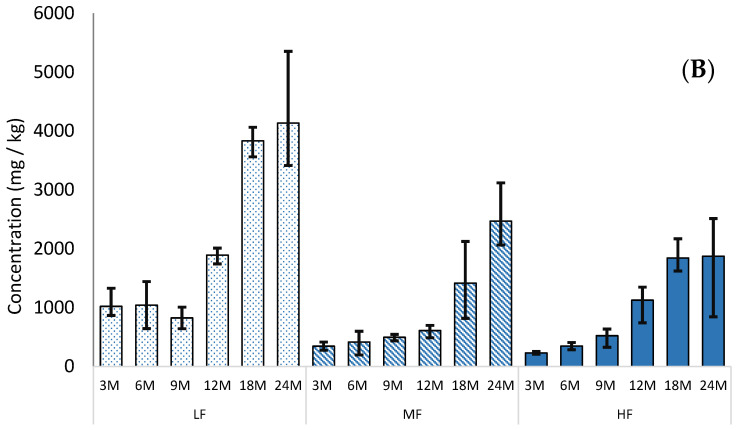
Comparison of mean dry weight concentrations (mg/kg) (mean N = 3) of (**A**) five amino acids in cooked (abbreviated to ‘C’, red bars) and uncooked (blue bars) Cheddars and (**B**) glutamic acid in Cheddars throughout ripening (3, 6, 9, 12, 18, and 24 months, respectively). The bar patterns indicate low (spotted), medium (striped), and high (solid colour) cheese, respectively. Error bars indicate the minimum and maximum range values.

Amino acid concentrations increased during ageing in the Cheddars. The low-fat cheeses contained higher concentrations of amino acids than in their MF or HF equivalents, as shown for glutamic acid in Figure 2B (Table S2). These findings agree with previous studies [18,19], although the rate of proteolysis has also been shown to be typically slower in low-fat cheeses [18,20,21]. This is related to a lower moisture-to-protein ratio in low-fat cheeses, which negatively affects the ease with which proteolytic microorganisms and enzymes can access their substrates. Amino acid formation occurs during proteolysis; therefore, the higher formation of amino acids in our low-fat cheeses could be attributed to a higher protein concentration rather than a faster rate of proteolysis.

During cooking, the amino acid concentrations decreased on a dry weight basis in all cheeses (see Figure 2A). Participation in the Maillard reaction and the formation of DKPs are likely to contribute to losses of amino acids during cooking. While the concentration of amino acids was higher in the low-fat cheese, there was also a greater loss of amino acids during cooking in the low-fat cheese (72% in LF, compared to 41% and 44% in MF and HF, respectively).

The rapid loss of amino acids during cooking in LF compared to HF may indicate Maillard reactions between amino acids and sugars; however, the loss of sugar in LF was much lower than in HF (see Section 3.4). Furthermore, the second part of this study (not shown) demonstrated that the majority of the volatile Maillard products were lower in the cooked LF than HF Cheddars. This suggests that the loss of amino acids in LF cheeses during cooking may occur through a different mechanism than the typical reaction with reducing sugars in the Maillard reaction.

Alternatively, another possible explanation for this difference is the physical effect of fat on the cooking process in cheese. Cheese structure is an amorphous casein network interspersed with globules of fat, moisture, and other components. During cooking, the fat globules coalesce and eventually pool into a free fat layer, which coats the cheese. In low-fat cheeses, there are fewer and smaller fat globules to interrupt the protein phase [21] and a lower moisture-to-protein ratio. This more continuous casein network may provide more opportunity for thermally induced reactions involving amino acids to occur. Furthermore, in low-fat cheeses, it has been shown that the absence of a free fat layer promotes rapid dehydration and browning [18,20,22], indicating the occurrence of thermally induced reactions.

The concentration of most amino acids was below their taste thresholds [23] in the mild Cheddars; however, isoleucine, aspartic acid, and glutamic acid were all above threshold in uncooked LF, and glutamic acid was also above threshold in uncooked MF and HF. In the cooked cheeses, glutamic acid was above the threshold in LF only. This suggests that glutamic acid may contribute to umami flavour in some cooked cheeses, although glutamic acid concentration decreased substantially during cooking.

### 3.3. γ-Glutamyl Peptides

**Figure 3:**
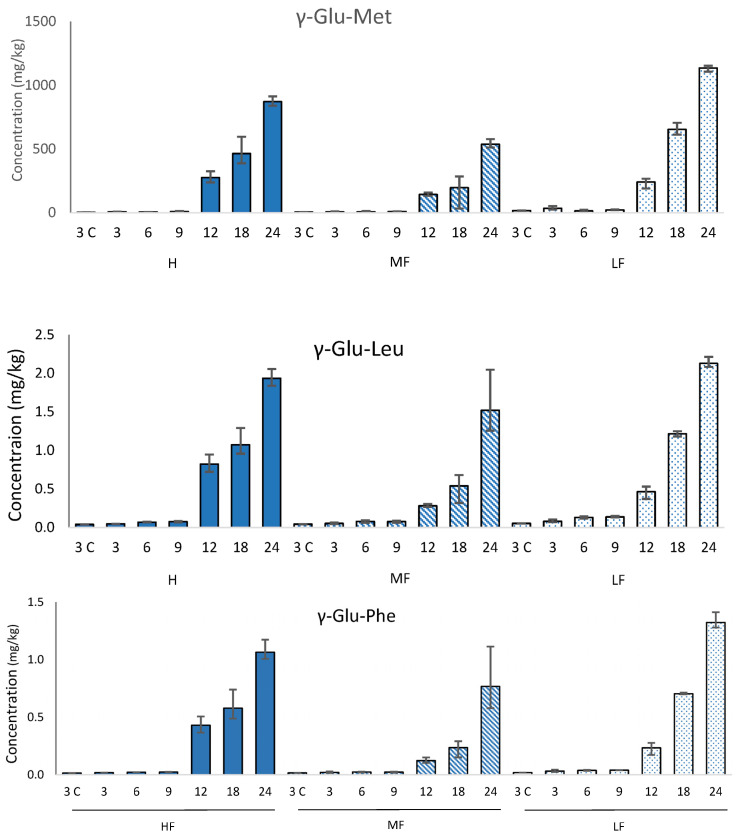
Concentration (mg/kg) of three γ-glutamyl peptides (mean N = 3) in high (HF, solid bars), medium (MF, striped bars), and low (LF, spotted bars) Cheddars throughout ripening (3, 6, 9, 12, 18, and 24 months, respectively) and cooking (3C) on a wet-weight basis. Error bars indicate the minimum and maximum range values.

Each of the γ-glutamyl peptides studied (γ-Glu-Glu, γ-Glu-Val, γ-Glu-Met, γ-Glu-Tyr, γ-Glu-Leu, γ-Glu-Phe) increased in concentration during aging; three examples are shown in Figure 3 (Table S3). This confirms previous work by Toelstede and Hofmann [6], which showed a higher concentration of kokumi peptides in 44-month-old gouda than in 4-month-old gouda. These changes are driven by the process of proteolysis during cheese ageing, in which long-chain proteins are broken down into smaller-chain peptides. The highest concentration was γ-Glu-Met, which also had the largest increase in concentration during ageing. The concentration of γ-Glu-Met at 12-month-old HF Cheddar was comparable to values reported by Toelstede and Hofmann [6] for ripened goats cheese and higher than values reported for 30-week-old Milner and 8-month-old Gruyère, suggesting that γ-Glu-Met formation during ripening progresses at a comparable speed in HF Cheddar to these cheeses. However, the highest concentration of γ-Glu-Met reported (1136 mg/kg in 24-month-old LF Cheddar) was higher than the highest concentration reported by Toelstede and Hofmann [6] in blue Shropshire. This high value is likely to be driven by the extended ageing period used during our study. The other γ-glutamyl peptides had lower concentrations than some aged cheeses reported in other studies. This difference is likely to be related to the different cultures used in the manufacture of the various cheeses. 

The concentration of γ-glutamyl peptides in the 24-month-old cheeses was not significantly different between the LF and HF samples. However, in the uncooked mild Cheddars (3 months old), only three γ-glutamyl peptides were detected (γ-Glu-Met, γ-Glu-Leu, and γ-Glu-Phe). In each case, there was significantly (*p* = 0.05) more γ-glutamyl peptide in the LF mild Cheddar than in the HF. This suggests that higher concentrations of γ-glutamyl peptides are generated during cheesemaking and the early stages of ripening in low-fat Cheddar; however, after more extensive ripening, the concentration is independent of the fat level in the cheese. The initial high concentration in LF cheese may be related to the higher protein concentration, while the subsequent more rapid formation of γ-glutamyl-peptides in HF cheese may be reflective of a higher rate of proteolysis. These results indicate that the overall kokumi character is likely to be similar in aged HF and LF cheeses alike.

Threshold values (in a water-soluble extract (WSE) from cheese) for γ-Glu-Met, γ-Glu-Glu, and γ-Glu-Leu were reported by Toelstede et al. [5]. Comparison of these values with the concentrations in our Cheddar demonstrates that γ-Glu-Met and γ-Glu-Leu are present above their thresholds in 24-month-old Cheddar, and γ-Glu-Met is above its threshold in uncooked and cooked 3-month-old Cheddar. The thresholds of these peptides in a cheese matrix have not yet been calculated; however, these data confirm previous studies that suggest it is likely that γ-glutamyl peptides play a role in aged cheese flavour. Furthermore, this study indicates that LF Cheddar generates γ-glutamyl peptides at a similar rate to normal Cheddar.

**Figure 4:**
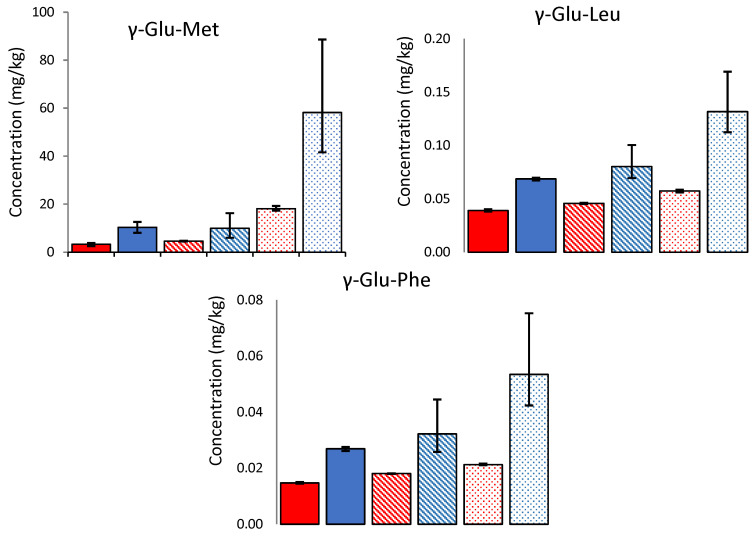
Concentration (mg/kg) of three γ-glutamyl peptides (mean N = 3) present in 3-month-aged Cheddars in both uncooked (blue) and cooked (red) samples, on a dry weight basis. The bar patterns indicate low (spotted), medium (striped), and high (solid colour) fat cheese, respectively. Error bars indicate the minimum and maximum range values.

In the cooked mild cheeses, the γ-glutamyl peptides were lower on a wet weight basis than their uncooked counterparts. Furthermore, comparison on a dry weight basis (to account for the loss of moisture in the cheese during cooking) indicates substantial losses of γ-glutamyl peptides during cooking (43–69%, see Figure 4). The concentrations were highest in the uncooked LF cheese but were more similar across the cooked cheeses. Previous literature has shown that dipeptides are susceptible to the Maillard reaction and can act as precursors of volatile compounds such as pyrazines [24]. While the concentration of γ-glutamyl dipeptides decreased during cooking, Glu-Met was the only one above its threshold in the uncooked WSE and was still above the threshold in the cooked WSE.

### 3.4. Sugars

**Figure 5:**
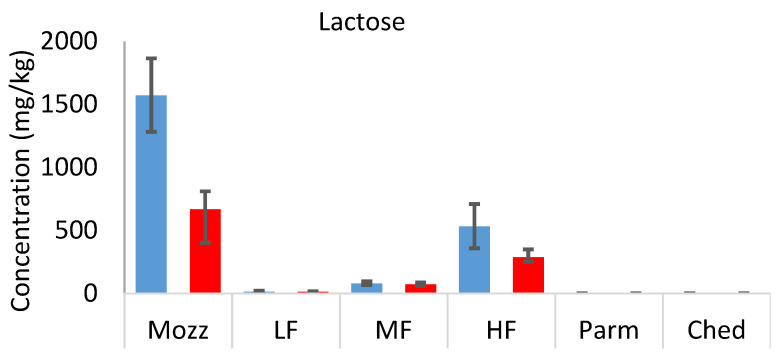

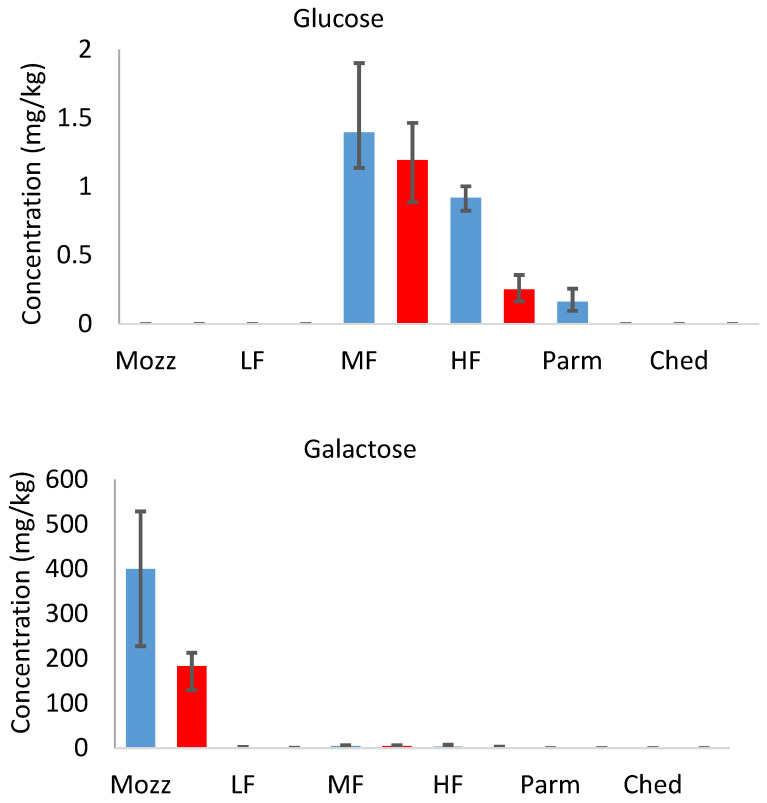
Dry weight concentrations (mg.kg) of lactose, glucose, and galactose in Parmesan (Parm), commercial mature Cheddar (Ched), mozzarella (Mozz), high-fat 3-month-aged Cheddar (HF), medium-fat 3-month-aged Cheddar (MF), and low-fat 3-month-aged Cheddar (LF), uncooked (blue bars) and cooked (red bars). Error bars show minimum and maximum range values.

In the full-fat uncooked cheeses, the concentration of sugars was inversely related to the typical ageing period [25] of each cheese (mozzarella > mild Cheddar > mature Cheddar and Parmesan). Figure 5 (Table S4) shows the concentration of lactose, glucose, and galactose in each cheese, both uncooked and cooked, on a dry weight basis. Lactose metabolism occurs during the early stages of cheese ripening and significantly decreases lactose concentration between fresh and aged cheeses [26]. The metabolism of lactose generates its two monosaccharide components, glucose and galactose. Much more galactose was detected in all samples than glucose; similar results have previously been reported [27]. When lactose is cleaved, the glucose moiety becomes a reactive leaving group, which is prone to further reactions, while the galactose produced is relatively less reactive. This is likely to contribute to the difference in concentration between glucose and galactose in uncooked cheeses.

In the cooked cheeses, similar concentrations of each of the sugars were detected compared to their uncooked counterparts (on a wet weight basis; see supplementary information). The concentration of lactose in each case was far below the threshold value [28], suggesting that lactose and the other sugars do not impart sweetness in cooked or uncooked mild Cheddar. This is expected, as the sweetness in cheese is more often attributed to amino acids (e.g., threonine, serine, glycine, and alanine) and salts (calcium and magnesium propanoates) [29]. However, these sugars are involved in the development of flavour during the cooking of cheese as they can act as precursors of other non-volatile and volatile compounds through the Maillard reaction and other mechanisms. A comparison of sugar concentrations on a dry weight basis (to account for moisture loss during cooking) demonstrates that as much as 58% of the sugars were lost during cooking.

The sugar concentration in the uncooked mild Cheddars decreased significantly in the order HF > MF > LF. A positive correlation between lactose concentration and fat content in cheese has been reported previously [21,30]. It is related to the rate of lactose metabolism during the cheesemaking and ageing process, as low-fat cheeses are prone to more rapid lactose metabolism. It may also indicate that the starter culture did not sufficiently metabolise lactose. The difference in galactose composition between LF, MF, and HF uncooked cheeses was not significant, indicating that further reactions of galactose may also happen more rapidly in low-fat Cheddar. The difference in lactose concentration (dry weight basis) between uncooked and cooked samples was also larger in the HF cheese, suggesting that more sugars are involved in Maillard or caramelisation reactions during cooking in high-fat cheese.

### 3.5. Organic Acids

**Figure 6:**
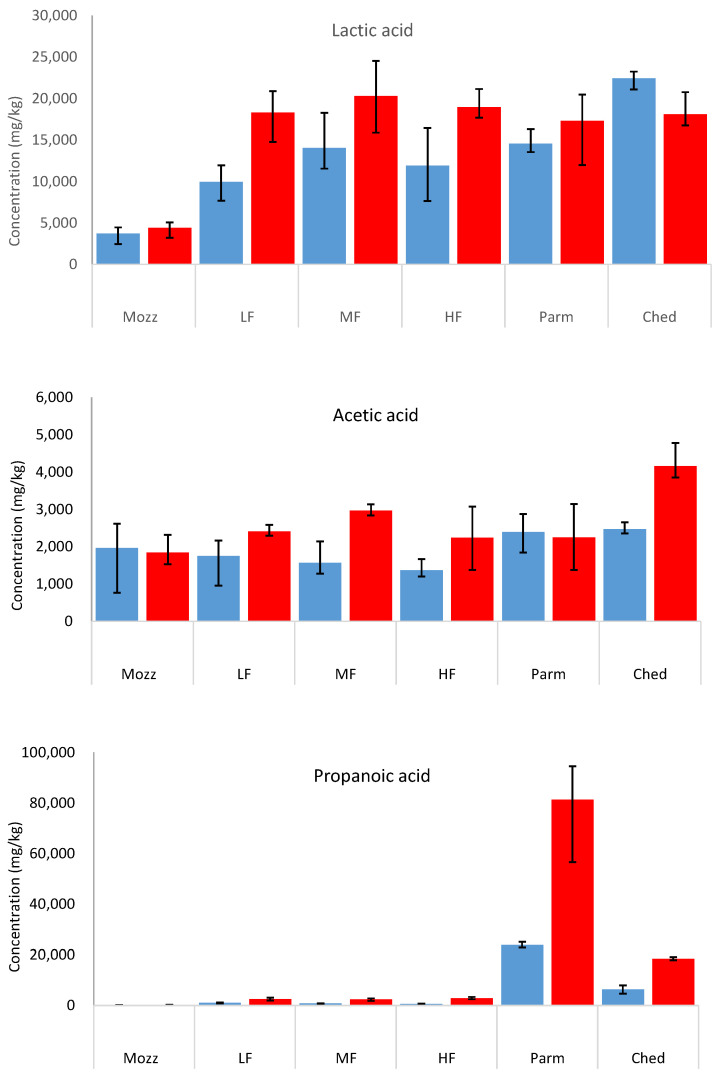
Mean dry weight concentrations (mg/kg) of lactic acid, acetic acid, and propanoic acid in Parmesan (Parm), mature Cheddar (Ched), mozzarella (Mozz), high-fat 3-month-aged Cheddar (HF), medium-fat 3-month-aged Cheddar (MF), and low-fat 3-month-aged Cheddar (LF), uncooked (blue bars) and cooked (red bars). Error bars show minimum and maximum range values.

Figure 6 (Table S5) shows dry weight comparisons of lactic, acetic, and propanoic acids in uncooked and cooked cheeses. All samples were substantially above the acetic acid and lactic acid thresholds for acidic taste in water [31]. In the uncooked cheeses, the lactic and propanoic acid concentrations were lowest in mozzarella. This may be due to mozzarella being a fresh (un-matured) cheese, as organic acid formation occurs during cheesemaking and maturation. Additionally, the high moisture content of mozzarella compared to the other cheeses studied has a diluting effect on the wet weight concentration of organic acids, as the dry weight concentrations in mozzarella were much closer to the other cheeses. 

The propanoic acid concentrations in the uncooked cheeses increased with the typical length of maturation (mozzarella < mild Cheddars < mature Cheddar < Parmesan). The lactic acid concentrations were also higher in the more aged cheeses; however, there was less difference in the lactic acid concentrations than in the propanoic acid concentrations. Similar results have been reported previously [25,32], which summarise how most residual lactose is metabolised rapidly after cheesemaking, such that lactic acid concentrations only increase marginally with longer maturation periods. This agrees with the results discussed in Section 3.4, in which the highest sugar concentrations were found in the youngest cheeses. In contrast, propanoic and butanoic acids are formed initially by the metabolism of lactose [26], by lipolytic processes that continue throughout the ageing period [32], and also via amino acid catabolism [33].

During cooking, each acid increased in concentration on a wet-weight basis. On a dry weight basis, the concentration of acetic acid was similar in the uncooked and cooked cheeses, while the lactic and propanoic acids increased in concentration. The conversion of sugars into small-chain organic acids during cooking occurs during the Maillard reaction [34]. However, the increase in the concentration of organic acids is higher on a molar basis than the loss of sugars discussed in Section 3.4. The additional formation of propanoic acid may occur via a lipid pathway.

## 4. Discussion

This study has demonstrated that there are changes in the concentration of selected non-volatile compounds in cheese during cooking. In some cases, the change in concentration during cooking altered which tastants were suprathreshold, which is likely to contribute to differences in flavour between uncooked and cooked cheese.

Sugars, amino acids, and γ-glutamyl dipeptides all decreased in concentration, which is likely due to their participation in thermally induced reactions such as the Maillard reaction. While the concentration of γ-glutamyl dipeptides decreased during cooking, their concentration was highly dependent on the extent of maturation of the cheese. Cooking caused up to 69% loss of γ-glutamyl dipeptides, while ageing from 3 to 24 months resulted in an over 120-fold increase. Cooked aged cheeses may therefore possess kokumi character due to these dipeptides. As with uncooked cheese, sugars were below their taste thresholds in cooked cheeses and unlikely to contribute directly to the flavour. Glutamic acid was suprathreshold in some uncooked cheeses but subthreshold in some of their cooked counterparts, suggesting that cooking may decrease the umami character of cheeses.

We report for the first time that DKPs increased in concentration during cooking in cheese and were above taste thresholds in some cooked cheeses. This suggests that bitterness may contribute more substantially to cooked cheese flavour than to uncooked cheese flavour, especially in cooked mature cheeses. Lactic and propanoic acid concentrations increased during cooking and were substantially above the acidic threshold in all samples, both cooked and uncooked. Acidic taste is likely to be as important to cooked cheese flavour as it is to uncooked cheese. Both DKP and organic acid formation are likely to be due to thermally induced reactions.

Our results suggest that fat may influence flavour formation during the cooking of cheese. The loss of amino acids was more rapid in LF than HF Cheddar, although the loss of sugars was more rapid in HF than LF cheese. Rapid dehydration and browning during cooking have been shown to occur in low-fat cheeses. This is attributed to the lack of a free fat layer coating low-fat cheese compared to regular fat cheeses during cooking.

In addition to the implications for the taste of cooked cheese, changes in the concentration of selected non-volatile compounds in cheese may have implications for the formation of volatiles during cooking through thermally induced reactions, including the Maillard reaction and caramelisation. Further work, which is yet to be published, will explore this topic by focusing on a comparison of volatile compounds in uncooked and cooked cheese.

This work has investigated the role of cooking on key non-volatiles in cheese. It is hoped this will give valuable insight for the dairy industry to inform the development of cheeses, especially low-fat variants, for use in cooked foods.

## Data Availability

All data are either provided in the manuscript or in the Supplementary Materials.

## Supplementary Materials

The following supporting information can be downloaded at: https://www.mdpi.com/article/10.3390/foods12203749/s1. Table S1: Quantitation of diketopiperazines (mg/kg) in cooked and raw cheeses (mean N = 3); Table S2: Quantitation of amino acids (mg/kg) in cooked and raw cheeses (mean N = 3); Table S3: Quantitation of γ-glutamyl peptides (mg/kg) in cooked and raw cheeses (mean N = 3); Table S4: Quantitation of sugars (mg/kg) in cooked and raw cheeses (mean N = 3); Table S5: Quantitation of organic acid (mg/kg) in cooked and raw cheeses (mean N = 3).
