# Supplementary material for: Characterisation of Cooked Cheese Flavour: Non-Volatile Components
Source: Foods. 2023 Oct 12;12(20):3749. doi: 10.3390/foods12203749 (PMC10606102; doi:10.3390/foods12203749)
Supplement: Supplementary file 1 [file foods-12-03749-s001.zip › foods-2614650-supplementary.pdf]

Characterisation of cooked cheese flavour: Part 1 – Non-volatile components

Supplementary Information

Rosa C. SULLIVAN [1,2], Samantha NOTTAGE[1], Fiyinfolu MAKINWA[1], Maria ORUNA-CONCHA[1], Colette C. FAGAN[1], Jane K. PARKER[1]*

[1] Department of Food and Nutritional Sciences, University of Reading, Whiteknights, Reading RG6 6DZ, UK

[2] Synergy Flavours Ltd, Hillbottom Road, Sands Industrial Estate, High Wycombe, HP124HJ, UK

*Corresponding author: Jane K. Parker, Department of Food and Nutritional Sciences, University of Reading, Whiteknights, Reading RG6 6AP, UK.

Telephone: +44 (0) 118 378 7455

E-mail: j.k.parker@reading.ac.uk

Table S1 Quantitation of diketopiperazines (mg/kg) in cooked and raw cheeses (mean N=3).

	c-Val-Pro	c-Leu-Pro	c-Ala-Pro	c-Pro-Pro
HF Cooked	32.7	16.1	20.6	65.0
HF Raw	ND	ND	ND	ND
HF24 Cooked	380	139	434	246
HF24 Raw	ND	ND	ND	ND
MF Cooked	44.0	27.7	26.2	68.8
MF Raw	ND	ND	ND	ND
LF Cooked	31.5	12.9	15.2	53.7
LF Raw	ND	ND	ND	ND
Mozz Cooked	33.8	21.5	ND	ND
Mozz Raw	7.4	5.5	ND	ND
Parm Cooked	153	91.3	160	32.7
Parm Raw	6.8	0.0	ND	ND
Ched Cooked	513	273	403	57.7
Ched Raw	3.5	0.0	ND	ND

ND indicates compounds which were not detected.

Table S2 Quantitation of amino acids (mg/kg) in cooked and raw cheeses (mean N=3)

	Ala		Gly		Val		Leu		Ile		Thr		Ser		Pro		Asn	
3M HF	77	F	43	E	218	FG	484	EFG	822	EF	36	EF	66	FG	72	EFG	187	DEFGH
3M MF	75	F	38	E	262	FG	480	EFG	815	EF	31	EF	39	G	63	EFG	144	EFGH
3M LF	290	BCD	174	B	573	CDEF	935	CDE	1591	CDEF	144	CDE	149	CDEFG	252	BCD	479	ABC
6M HF	97	F	43	E	274	EFG	635	DEFG	1076	DEF	55	EF	79	EFG	57	FG	215	CDEFGH
6M MF	91	F	38	E	264	FG	608	EFG	1031	DEF	47	EF	57	FG	60	EFG	208	DEFGH
6M LF	185	CDEF	90	CDE	550	CDEF	854	CDEFG	1447	CDEF	128	CDEF	149	CDEFG	170	BCDEFG	404	ABCDE
9M HF	139	DEF	71	DE	399	DEFG	863	CDEFG	614	F	72	EF	99	EFG	109	DEFG	345	BCDEFG
9M MF	130	EF	52	E	382	EFG	824	CDEFG	1005	DEF	69	EF	97	EFG	106	DEFG	299	CDEFGH
9M LF	177	CDEF	93	BCDE	518	CDEFG	829	CDEFG	1408	CDEF	125	CDEF	140	CDEFG	174	BCDEFG	397	ABCDEF
12M HF	121	EF	73	DE	469	DEFG	843	CDEFG	1431	CDEF	91	DEF	108	DEFG	153	BCDEFG	264	CDEFGH
12M MF	156	DEF	83	CDE	451	DEFG	902	CDEF	1165	DEF	96	DEF	140	CDEFG	128	CDEFG	325	BCDEFGH
12M LF	320	BC	158	BC	914	BC	1472	BC	2498	ABCD	228	BC	260	BCDE	303	BC	577	AB
18M HF	256	BCDE	151	BCD	803	BCD	1455	BC	2468	ABCD	202	BCD	300	BCD	222	BCDEF	444	ABCD
18M MF	217	BCDEF	107	BCDE	722	BCDE	1360	BCD	2307	BCDE	154	BCDE	242	BCDEF	173	BCDEFG	390	ABCDEF
18M LF	567	A	300	A	1613	A	2381	A	4040	A	469	A	634	A	564	A	637	A
24M HF	288	BCD	184	B	920	BC	1727	AB	2827	ABC	229	BC	322	BC	235	BCDE	415	ABCDE
24M MF	345	B	179	B	1062	B	1743	AB	2960	ABC	273	B	410	B	325	B	431	ABCDE
24M LF	543	A	295	A	1533	A	2290	A	3897	AB	468	A	640	A	573	A	289	CDEFGH
C 3M HF	65	F	33	E	82	G	218	FG	372	F	17	F	32	G	47	FG	85	GH
C 3M MF	64	F	25	E	73	G	172	G	293	F	15	F	24	G	41	G	73	H
C 3M LF	99	F	44	E	133	G	228	FG	388	F	37	EF	50	G	76	DEFG	129	FGH

Table S2 Cont

	Asp		Met		Glu		Phe		Orn		Lys		His		Tyr		Trp	
3M HF	70	C	25	D	231	DE	368	GHIJ	76	E	167	DEF	27	E	83	FGH	4.2	DE
3M MF	52	C	45	D	345	DE	387	FGHIJ	62	E	107	DEF	3.4	E	75	FGH	6.5	DE
3M LF	179	C	140	CD	1022	CDE	830	CDEF	345	BC	635	BC	46	DE	179	EFG	14.1	CDE
6M HF	82	C	40	D	347	DE	391	FGHIJ	102	E	118	DEF	0	E	124	FGH	1.2	E
6M MF	90	C	61	D	415	DE	385	FGHIJ	42	E	39	F	0	E	61	GH	0	E
6M LF	164	C	100	CD	1042	CDE	646	CDEFGHI	135	CDE	155	DEF	0	E	138	FGH	6.4	DE
9M HF	114	C	148	CD	523	DE	572	DEFGHIJ	74	E	111	DEF	0	E	152	FGH	6.4	DE
9M MF	132	C	89	CD	496	DE	508	EFGHIJ	104	E	147	DEF	0	E	135	FGH	2.1	DE
9M LF	144	C	169	CD	824	CDE	647	CDEFGH	163	BCDE	202	CDEF	0	E	160	EFGH	12.1	CDE
12M HF	127	C	143	CD	1128	CDE	542	EFGHIJ	125	DE	237	CDEF	35	E	220	DEF	18.4	CDE
12M MF	139	C	106	CD	610	CDE	561	DEFGHIJ	113	DE	178	DEF	23	E	166	EFGH	7.2	DE
12M LF	350	BC	359	B	1891	BC	1041	BC	355	B	490	BCD	55	DE	303	CDE	45.2	BC
18M HF	290	BC	239	BC	1843	BC	852	CDE	225	BCDE	501	BCD	181	BC	352	BCD	34.7	CD
18M MF	277	BC	142	CD	1416	BCD	762	CDEFG	192	BCDE	462	BCDE	128	CD	302	CDE	11.3	DE
18M LF	981	A	647	A	3832	A	1552	A	647	A	1219	A	315	A	502	A	91.6	A
24M HF	252	BC	373	B	1873	BC	1000	BCD	199	BCDE	471	BCDE	228	B	457	AB	68.9	AB
24M MF	559	B	400	B	2468	B	1005	BCD	320	BCD	761	B	225	B	401	ABC	44.8	BC
24M LF	1260	A	664	A	4133	A	1397	AB	615	A	1295	A	369	A	502	A	96.8	A
C 3M HF	42	C	28	D	68	E	192	IJ	45	E	51	F	4.5	E	36	GH	4.5	DE
C 3M MF	34	C	21	D	113	E	163	J	59	E	67	EF	5.3	E	29	H	2.3	DE
C 3M LF	60	C	43	D	206	DE	226	HIJ	81	E	128	DEF	17	E	46	GH	6.4	DE

3M = 3 months aged, 6M = 6 months aged, 9M = 9 months aged, 12M = 12 months aged, 24M = 24 months aged, C 3M = cooked 3 months aged. LF, MF and HF = low, medium and high fat cheddar respectively. Letters A-J indicate significance based on Tukey's HSD: within each column, samples with the same letter were not significantly different (p=0.05).

Table S3 γ - Quantitation of γ-glutamyl peptides (mg/kg) in cooked and raw cheeses (mean N=3)

	γ-Glu-Glu	γ-Glu-Val	γ-Glu-Met	γ-Glu-Tyr	γ-Glu-Leu	γ-Glu-Phe
3M HF	0.000 e	0.000 e	2.1	0.000 f	0.014 i	0.005 f
3M MF	0.000 e	0.000 e	2.0	0.000 f	0.016 i	0.006 f
3M LF	0.004 de	0.001 e	11.6	0.000 f	0.026 i	0.011 f
6M HF	0.000 e	0.001 e	1.4	0.000 f	0.021 i	0.007 f
6M MF	0.000 e	0.002 e	1.9	0.000 f	0.024 i	0.007 f
6M LF	0.000 e	0.012 e	4.7	0.003 ef	0.043 hi	0.013 f
9M HF	0.000 e	0.003 e	2.7	0.000 f	0.022 i	0.007 f
9M MF	0.000 e	0.005 e	2.6	0.000 f	0.023 i	0.007 f
9M LF	0.000 e	0.012 e	7.0	0.002 f	0.046 ghi	0.013 f
12M HF	0.045 c	0.060 cd	82.1	0.038 d	0.245 ef	0.128 de
12M MF	0.000 e	0.016 de	44.5	0.010 ef	0.087 ghi	0.038 f
12M LF	0.000 e	0.037 cde	80.4	0.019 e	0.155 fgh	0.078 ef
18M HF	0.044 c	0.069 c	138.4	0.048 cd	0.319 de	0.173 cd
18M MF	0.019 d	0.039 cde	61.1	0.018 e	0.168 fg	0.073 ef
18M LF	0.058 bc	0.115 b	218.4	0.055 bc	0.405 cd	0.235 c
24M HF	0.072 ab	0.122 b	260.3	0.095 a	0.577 b	0.318 b
24M MF	0.045 c	0.124 b	166.8	0.066 b	0.472 bc	0.238 c
24M LF	0.087 a	0.202 a	379.3	0.096 a	0.710 a	0.442 a
C 3M LF	0.000 e	0.000 e	3.6	0.000 f	0.011 i	0.004 f
C 3M MF	0.000 e	0.000 e	0.9	0.000 f	0.009 i	0.004 f
C 3M HF	0.000 e	0.000 e	0.7	0.000 f	0.008 i	0.003 f

3M = 3 months aged, 6M = 6 months aged, 9M = 9 months aged, 12M = 12 months aged, 24M = 24 months aged, C 3M = cooked 3 months aged. LF, MF and HF = low, medium and high fat cheddar respectively. Letters a- i indicate significance based on Tukey's HSD: within each column, samples with the same letter were not significantly different (p=0.05).

Table S4 Quantitation of sugars (mg/kg) in cooked and raw cheeses (mean N=3).

	Lactose		Glucose		Galactose	
Cheddar Cooked	0.19	d	nd	c	0.001	c
Cheddar Raw	nd	d	nd	c	nd	c
HF Cooked	28.8	cd	0.025	c	0.24	c
HF Raw	53.1	bc	0.09	b	0.37	c
LF Cooked	1.31	d	nd	c	0.07	c
LF Raw	1.23	d	nd	c	0.16	c
MF Cooked	7.24	d	0.12	ab	0.46	c
MF Raw	7.96	d	0.14	ab	0.45	c
Mozzarella Cooked	66.7	d	nd	c	18.34	b
Mozzarella Raw	157	a	nd	c	40.00	a
Parmesan Cooked	0.06	d	nd	c	nd	c
Parmesan Raw	0.00	d	0.016	c	0.002	c

Letters a-d indicate significance based on Tukey's HSD: within each column, samples with the same letter were not significantly different (p=0.05).

Table S5 Quantitation of organic acid (mg/kg) in cooked and raw cheeses (mean N=3).

	Citric acid		Malic Acid		Lactic acid		Acetic acid		Propanoic acid	
Raw Cheddar	10.4	b	32.2	a	448	a	49.3	ab	126	bc
Cooked Cheddar	6.03	bcd	3.64	def	362	abc	83.2	a	368	bc
Raw Parmesan	1.56	d	3.07	ef	291	abc	47.7	b	476	b
Cooked Parmesan	2.08	d	4.39	cdef	346	abc	45.0	b	1625	a
Raw Mozzarella	9.26	bcd	13.9	b	74	d	39.3	b	2.31	c
Cooked Mozzarella	16.82	a	1.45	f	88	d	36.8	b	2.73	c
Raw LF	4.34	bcd	2.03	ef	198	cd	35.0	b	20.4	c
Cooked LF	3.50	cd	4.66	cdef	366	abc	48.1	b	50.8	c
Raw MF	3.16	cd	6.78	cde	280	abc	31.4	b	16.2	c
Cooked MF	5.02	bcd	8.64	c	406	abc	59.3	ab	47.7	c
Raw HF	2.54	d	6.62	cde	237	bcd	27.4	b	10.9	c
Cooked HF	6.13	bcd	7.90	cd	379	ab	44.9	b	56.2	c

Letters a-f indicate significance based on Tukey's HSD: within each column, samples with the same letter were not significantly different (p=0.05).
ND indicates compounds which were not detected.
